# Acceptability in the Older Population: The Importance of an Appropriate Tablet Size

**DOI:** 10.3390/pharmaceutics12080746

**Published:** 2020-08-08

**Authors:** Thibault Vallet, Hugues Michelon, Mine Orlu, Yogini Jani, Patrick Leglise, Sandra Laribe-Caget, Matthieu Piccoli, Aurélie Le Fur, Fang Liu, Fabrice Ruiz, Vincent Boudy

**Affiliations:** 1 ClinSearch, 110 Avenue Pierre Brossolette, 92240 Malakoff, France; thibault.vallet@clinsearch.net (T.V.); aurelie.lefur@clinsearch.net (A.L.F.); 2 Pharmacy department, Hôpital Sainte-Périne, Groupe Hospitalier Universitaire AP-HP.Paris-Saclay, Assistance Publique-Hôpitaux de Paris (AP-HP), 11 rue Chardon-Lagache, 75016 Paris, France; hugues.michelon@aphp.fr; 3 Department of Pharmaceutics, UCL School of Pharmacy, 29–39 Brunswick Square, London WC1N 1AX, UK; m.orlu@ucl.ac.uk; 4 Centre for Medicines Optimisation Research and Education, University College London Hospitals NHS Foundation Trust, London NW1 2BU, UK; y.jani@ucl.ac.uk; 5 Pharmacy department, Hôpital Joffre Dupuytren, Hôpitaux Universitaires Henri Mondor, Assistance Publique-Hôpitaux de Paris (AP-HP), 1 rue Eugène Delacroix, 91210 Draveil, France; patrick.leglise@aphp.fr; 6 Pharmacy department, Hôpital Rothschild, Hôpitaux Universitaires Est Parisien, Assistance Publique-Hôpitaux de Paris (AP-HP), 5 Rue Santerre, 75012 Paris, France; sandra.laribe-caget@aphp.fr; 7 Hôpital Broca, Hôpitaux Universitaires Paris Centre, Assistance Publique-Hôpitaux de Paris (APHP), 54–56 Rue Pascal, 75013 Paris, France; matthieu.piccoli@aphp.fr; 8 Department of Clinical and Pharmaceutical Sciences, School of Life and Medical Sciences, University of Hertfordshire, Hatfield AL10 9AB, UK; f.liu3@herts.ac.uk; 9 Unité de R&D Galénique, Agence Générale des Equipements et Produits de Santé (AGEPS), Assistance Publique-Hôpitaux de Paris (AP-HP), 7 rue du Fer À Moulin, 75005 Paris, France; vincent.boudy@aphp.fr; 10 UTCBS, Chemical and Biological Technologies for Health Laboratory, CNRS, INSERM, Université de Paris, 75006 Paris, France

**Keywords:** medicine, SODF, acceptability, older population, formulation, size, CAST (ClinSearch acceptability score test)

## Abstract

Presenting many advantages, solid oral dosage forms (SODFs) are widely manufactured and frequently prescribed in older populations regardless of the specific characteristics of patients. Commonly, patients with dysphagia (swallowing disorders) experience difficulties taking SODFs, which may lead to non-adherence or misuse. SODF characteristics (e.g., size, shape, thickness) are likely to influence swallowability. Herein, we used the acceptability reference framework (the ClinSearch acceptability score test (CAST))—a 3D-map juxtaposing two acceptability profiles—to investigate the impact of tablet size on acceptability. We collected 938 observer reports on the tablet intake by patients ≥65 years in hospitals or care homes. As we might expect, tablets could be classified as accepted in older patients without dysphagia (*n* = 790), while not in those with swallowing disorders (*n* = 146). However, reducing the tablet size had a significant impact on acceptability in this subpopulation: tablets <6.5 mm appeared to be accepted by patients with swallowing disorders. Among the 309 distinct tablets assessed in this study, ranging in size from 4.7 to 21.5 mm, 83% are ≥6.5 mm and consequently may be poorly accepted by institutionalized older people and older inpatients suffering from dysphagia. This underlines the need to develop and prescribe medicines with the best adapted characteristics to reach an optimal acceptability in targeted users.

## 1. Introduction

The proportion of the world’s population over 65 years is expected to double between 2019 and 2050, from 1 in 11 people in the world to 1 in 6 [1]. This growing population has particular needs that require special considerations concerning healthcare due to changes in a number of closely related factors, such as the deterioration of physical and cognitive abilities, multimorbidity, polypharmacy, and/or frailty [2]. Older people, the main users of medicines, are also a vulnerable population that faces increasing risks regarding medicine use. As well as drug–drug interactions, which are a common problem due to polypharmacy, inappropriate dosage forms may negatively impact patient adherence. Furthermore, the consequent misuse may result in medication errors and alterations to posology that could reduce effectiveness or lead to drug related adverse effects. The acceptability of medicines—defined as the overall ability and willingness of the patient to use, and of any caregivers to administer, the medicine as intended—is thus of the utmost importance in this vulnerable population [2].

Dysphagia is one of the most common age-related alterations in the elderly. About 30% to 40% of the older patients in institutions are affected by swallowing disorders [3]. This comorbidity has a negative impact on the ingestion of food and liquids, and thus on oral medication intake. Solid oral dosage forms (SODFs) are widely manufactured due to many advantages such as dosage accuracy, stability, storage, portability, ease of use and the possibility of combining several active substances. Consequently, tablets and capsules are the most common form of medicines, particularly in older patients who have high prescription rates. However, the negative impact of swallowing alteration on oral intake of such formulations is well-known [4,5]. Such impairments may lead to pain, choking, or serious life-threatening aspiration pneumonia during the oropharyngeal swallowing phase [6,7]. End-user alterations of dosage forms prior to administration, such as opening a capsule or crushing/splitting a tablet, is a widespread practice which can lead to adverse drug reactions both for patients and caregivers [8,9]. Crushing a prolonged release tablet could seriously compromise the pharmacokinetics and/or pharmacodynamics of the product [10]. Such unlicensed use may alter the efficiency of the treatment, or even induce toxic effects for the patients as well as for the caregivers [4,11,12].

Tablet characteristics such as the size, shape, and surface coating, are likely to influence the patient’s ability to swallow and consequently, the medicine’s acceptability [13]. Most patients taking daily SODFs have already experienced difficulty swallowing tablets, and the excessive size is one of the main related complaints [14,15,16].

Herein, we investigated the acceptability of tablets in the older population, aiming to better understand how tablet size affects the acceptability in older patients with a swallowing alteration.

## 2. Materials and Methods

To perform our explorations, we used the CAST (ClinSearch Acceptability Score Test^®^), which is briefly described in this section. This data driven approach, initially developed and validated in pediatrics [17,18] and then transposed to the elderly [19], provides relevant knowledge on medicine features that best fit different subpopulations of patients [20,21,22].

### 2.1. Data Collection

The data were collected in a multicenter, prospective, cross-sectional and strictly observational study conducted in France and the United Kingdom (UK) between October 2016 and November 2019. Twelve care homes and elderly care wards of nine hospitals were involved in the study.

After checking with the clinical team that there are no reasons why a participant should not be approached, eligible individuals—patients 65 years and over taking any medicine—were approached by a member of the research team in accordance with local regulations. In France, the patients (and/or legal representatives) were informed and included in this observational study if they did not express their verbal opposition. In the UK, written informed consent was obtained. Once enrolled in the study, a standardized questionnaire was completed by a trained member of the site study team observing the use of the very first medicine due to be administered at the next medication round for each participant. As such, any potential bias caused by the prior or co-administration of other medicines was avoided. Any medicinal products could be assessed, with the exception of infusions in which a catheter was already present, as the insertion of such a device was considered as part of the acceptability.

Some elderly patients are unable to provide reliable and valid self-evaluations due to physical and cognitive impairments. Consequently, in order to standardize the data collection in the older population, we used observer-reported outcomes including only those events or behaviors that can be observed, as is encouraged for cognitively impaired patients [23]. Following nurses during their medication rounds, the researchers reported their observations for the first medicine taken by each patient involved in the study. These included:

- the results of intake (the required dose fully, partly or not taken at all);
- the patient reaction during the administration using a 3-point hedonic face scale (positive, neutral or negative reaction);
- the time needed to prepare (from opening any packaging to having a required dose of medication ready to use, including all handling and modifications) and to administer the required dose of medication (from a required dose of medication ready to use to the end of the intake). The sum of the times of preparation and administration were both recorded with a timer and reported using 10 s intervals, classified as short (20 s and less), medium (from 30 s to 1 min), or long (more than 1 min).

Any of the following methods used to ease/achieve administration were also reported (binary variables with two possible values: use or not):

- dividing the intake of a dose which cannot be taken as a whole;
- altering the intended use (modifying the dosage form such as opening a capsule or crushing a tablet; using another route/mode of administration);
- using food/drink to mask a bad taste or ease swallowing;
- using a device not provided;
- using restraint (the patient had to be made to take it).

In addition, the researchers filled in the exact name of the medicine taken by the patient, information on the context of use (e.g., the place of medicine administration and the person in charge), as well as certain characteristics of both the patient (e.g., age, sex, swallowing disorders) and the treatment (e.g., the required dose, co-prescribing) recorded from the patient’s medical record.

Subsequently, information on the medicines were collected from the summary of product characteristics (SmPC). If the physical attributes of tablets, e.g., size, were not available in the SmPC, an inquiry was sent to the marketing authorization holder by email. For cases in which the manufacturer refused to share data or failed to reply, the physical attributes were obtained from TICTAC Communications Ltd. in the UK, and the Centre Antipoison et de Toxicovigilance (CAPTV) du Centre Hospitalier Régional Universitaire (CHRU) de Nancy in France. Size was defined as the largest dimension of the tablet referring to the length of oval or oblong tablets or the diameter of round tablets [24].

### 2.2. Data Analysis

Each evaluation of one medicinal product, taken by one patient, corresponded to a particular combination of an observed measure (e.g., fully taken) for each of the eight aforementioned observational variables (e.g., result of the intake) which describe the many aspects of acceptability. In total, 2004 evaluations were collected. A multivariate analysis mined this large set of standardized evaluations to summarize the main information into an intelligible tool: the acceptability reference framework.

The key relationships between the observed measures were visualized in a low-dimensional Euclidean space: the 3D acceptability map. A multifactorial process, multiple correspondence analysis (MCA), established the relationships between the observed measures that were often selected together in the evaluations, such as a “short time” and “positive reaction” or “use food/drink” and “use divided dose”. Such measures, as well as dots representing the evaluations completed in a similar manner, converged on the acceptability map. Thus, proximity on the map reflected a similarity. The three dimensions illustrated by the map summarized those associations and dissociations that most contributed to the total variance of the dataset (inertia). The first axis is the most important dimension, resuming 20.4% of the inertia, the second axis the next most important, summarizing 13% of the inertia, while the third dimension resumed a further 9.9% of the observed variations. Thus, the map highlighted the major information in terms of medicine acceptability variations.

Afterward, a clustering process gathered the most similar evaluations—the closest on the map—into two coherent clusters. The positive observations naturally emerged in the first cluster, defining the “positively accepted” profile, while all the negative observations were over-represented in the second cluster, defining the “Negatively accepted” profile. The profiles were materialized by a green and a red area on the map.

The evaluations of tablet intake were successively partitioned into two subgroups according to the patient’s ability to swallow and the size of the tablet taken:

- the older patients without swallowing disorders (SD−) and the older patients with swallowing disorders (SD+) who had taken tablets, regardless of their size;
- the older patients SD+ had taken tablets smaller than a given threshold and the older patients SD+ had taken tablets which are equal and larger than the threshold. Size thresholds increasing by steps of 0.5 mm were explored from 6 mm to 10 mm.

In each case, both subgroups of interest were positioned on the map, at the barycenter of their evaluations. If a barycenter, along with the entire 90% confidence ellipsis surrounding it, belonged to the green area of the map, the subgroup could be classified as accepted (see the video abstract for an illustration of the mapping, clustering, and scoring processes). A minimum of 30 evaluations are required to obtain a reliable acceptability score.

In each case, statistical tests were used to assess the significance of the differences observed between the two subgroups of interest in terms of patients’ characteristics, products’ features and measures composing the acceptability scores. For the categorical variables, when there was a minimum expectation of 5 for 80% of the cells without any null expectation, Pearson’s chi-squared test was used; alternatively, Fisher’s exact test was used. A Student’s *t*-test was used for the quantitative variable, i.e., to compare the mean tablet sizes evaluated between the two subgroups of patients, those with and without reported swallowing difficulties.

## 3. Results

### 3.1. Patients and Medicines

Among all the 2004 evaluations of any dosage forms that gave rise to the acceptability reference framework, there were 938 evaluations of tablet intake related to 309 distinct medicinal products. Among which, 61.5% of these products were assessed only once, while five of these products (1.6%) were assessed 30 times or more.

These 938 evaluations were mostly collected in France (81.3%) and in hospitals (70%). The mean age of the patients was 86 years (7.3), the minimum was 65, the maximum was 104, and 67% were women. Swallowing disorders diagnosed a priori have been reported from the patient’s medical record in 15.6% of these evaluations (two missing data). According to the second level (therapeutic subgroup) of the Anatomical Therapeutic Chemical (ATC) classification system, the medication was a drug from the antithrombotic agents pharmacological group for 21% of evaluations, the psychoanaleptics group for 11.5% (71% antidepressants and 29% anti-dementia drugs), and the psycholeptics group for 11.1% (80% anxiolytics, 18% antipsychotics, and 2% hypnotics and sedatives).

The 309 distinct medicinal products formulated as a tablet ranged from 4.7 to 21.5 mm. The mean size was 9.4 mm (3.8). The data were not available for 23 evaluations (20 distinct tablets).

### 3.2. Acceptability

**Figure 1:**
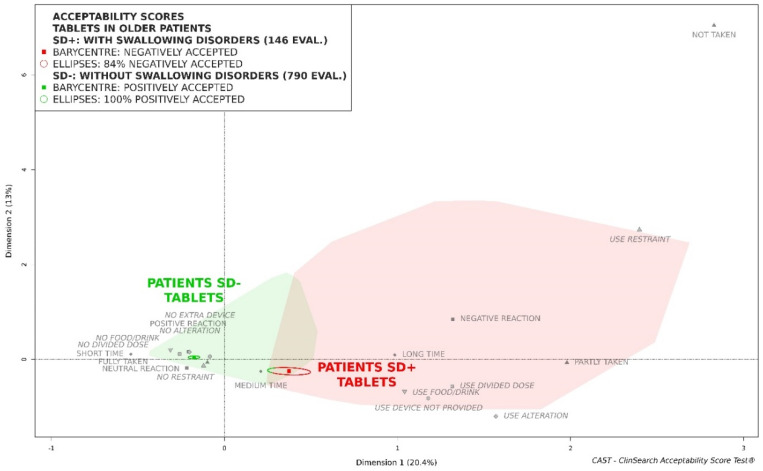
Acceptability profiles of tablets in the older patients with swallowing disorders (SD+) and without swallowing disorders (SD−).

Considered as a whole, tablets could be classified as accepted in the older population. Indeed, the barycenter of the 938 evaluations of tablet intake, along with the entire confidence ellipses surrounding it, was fully located in the green area of the acceptability map (see the video abstract). However, it appeared that swallowing disorders negatively impact tablet acceptability (Figure 1). Indeed, tablets were fully located in the green area of the map for patients without swallowing disorders, while they could not be considered as accepted in patients with a swallowing alteration. Supplementary Table S1 presents the characteristics of the patients and the medicines for both subgroups of patients. The patients with a swallowing alteration appeared to be much more fragile than their counterparts without swallowing disorders. Indeed, according to the Pearson’s chi-squared tests, there were significant differences in term of memory disorders (*p* = 0.002), muscular or rheumatologic disorders of the upper limbs (*p* < 0.001), and caregivers’ involvement in administration (*p* < 0.001). However, the two subgroups of patients appeared to be quite similar in terms of their demographics—there was only a slight difference in age groups distribution—, setting, and medicine: sex (*p* = 0.46), age subgroup (*p* = 0.026), place (*p* = 0.41), country (*p* = 0.93), and therapeutics of medicines (*p* = 0.18). According to the Student’s *t*-test, there was no difference in terms of tablet size (*p* = 0.34).

Supplementary Table S2 presents the size of the 78 medicinal products formulated as a tablet that were assessed in patients with a swallowing alteration. The information was not available for five evaluations (five distinct tablets). Considering the insufficient number of evaluations for each size (*n* < 30), we describe value ranges from the smallest to the largest tablet sizes.

**Figure 2:**
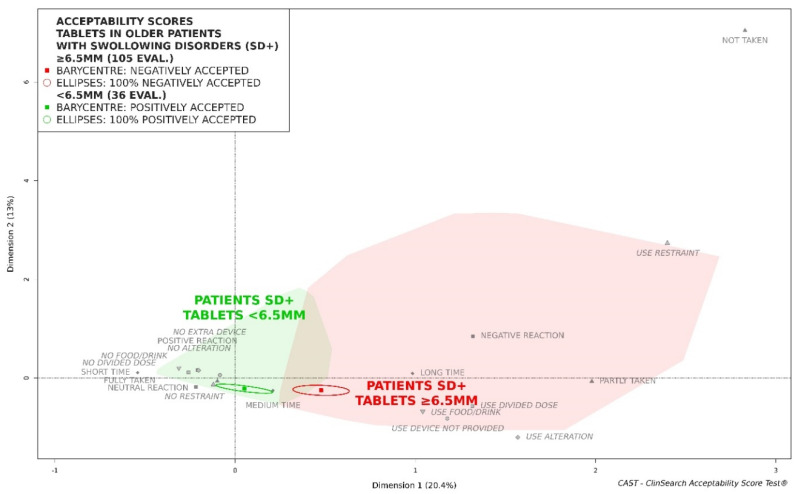
Acceptability profiles of tablets smaller than 6.5 mm and tablets which are 6.5 mm and larger in the older patients with swallowing disorders (SD+).

In the first quartile, tablets were smaller than 6.5 mm for 25% of the evaluations in those patients. Figure 2 highlights that those tablets smaller than 6.5 mm appeared to be positively accepted in this subpopulation, while the tablets which are 6.5 mm and larger were fully located in the red part of the map.

The two subgroups of patients with swallowing difficulties—those who had taken tablets smaller than 6.5 mm, and those who had taken tablets which are 6.5 mm and larger—appeared to be quite similar. Supplementary Table S3 presents the characteristics of the patients and the medicines for both subgroups of patients. According to the Pearson’s chi-squared tests, there were no significant differences in term of sex (*p* = 0.68), age subgroups (*p* = 0.64), memory disorders (*p* = 0.21), muscular or rheumatologic disorders of the upper limbs (*p* = 0.91), polymedications (*p* = 0.36), person in charge of administration (*p* = 0.98), place (*p* = 0.2), or country (*p* = 0.47). The sole difference was in terms of the administration timing, according to Fisher’s exact test (*p* = 0.007), as the proportion of smaller tablets taken in the morning appeared to be larger.

For both subgroups of patients, the largest percentage of medications were drugs from the antithrombotic agent pharmacological groups: 25% and 22% for the larger and smaller tablets, respectively. Other groups of medications that were represented included psycholeptics (17%) and psychoanaleptics (14%) for the larger tablets, and drugs used in diabetes (22%) and antianemic preparations (19%) for the smaller tablets.

These differences in acceptability scores reflect the differences observed for most of the constituting variables (Table 1). Negative observations were reported in patients with swallowing disorders taking a tablet of ≥6.5 mm more often than for their counterparts taking smaller tablets. The observers reported a negative reaction, a divided intake of the required dose, and the use of alterations significantly more often for the larger tablets than for the smaller ones. Furthermore, the required dose of drug seemed to be more often fully taken, and in a shorter time, for the smaller tablets (non-significant).

**Figure 3:**
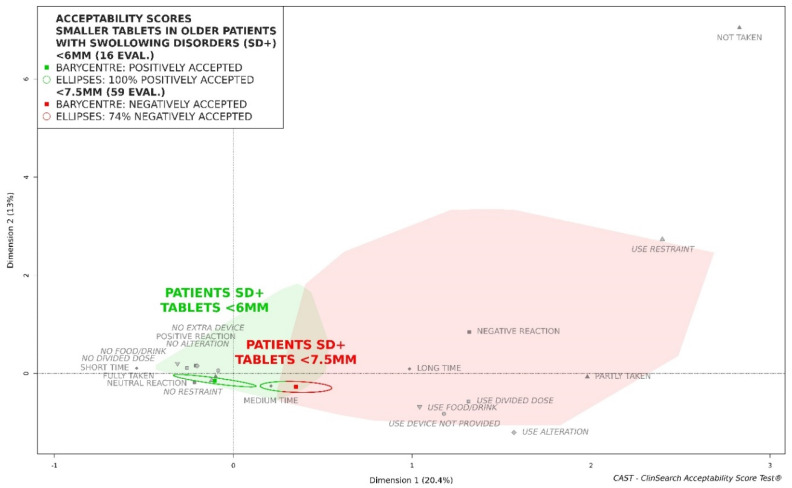
Acceptability profiles of tablets smaller than 6 mm and tablets smaller than 7.5 mm in the older patients with swallowing disorders (SD+).

This threshold of <6.5 mm sized tablets is the largest for which the barycenter and its entire confidence ellipse remain completely within the positively accepted cluster. The barycenter of the smaller tablets’ evaluations appeared to be located closest to the ideal position on the left side of the map when reducing the threshold to 6 mm (Figure 3). However, considering the insufficient number of evaluations (*n* = 16), this may only be described as an acceptability tendency. Conversely, upon increasing the size threshold, 0.5 mm by 0.5 mm, the barycenter of the smaller tablets’ evaluations moved to the right with part of their ellipses overlapping the negative area of the map: 8% of ellipses were in the red zone for <7 mm (*n* = 38), 74% for <7.5 mm (*n* = 59), 75% for <8 mm (*n* = 60), 79% for <8.5 mm (*n* = 72), 78% for <9 mm (*n* = 97), 79% for <9.5 mm (*n* = 104), and 83% for <10 mm (*n* = 109). The barycenter shifted toward the negative area of the map from a threshold of <7.5 mm (Figure 3).

## 4. Discussion

Using a validated multivariate approach integrating the many aspects of acceptability [19], our results confirmed that tablet size affects acceptability in older patients with swallowing disorders. This study objectively demonstrates that reducing the size of tablets has a positive impact on acceptability in this subpopulation of patients. Tablets smaller than 6.5 mm appeared to be positively accepted by those patients, while the largest tablets were located in the negative zone of the acceptability reference framework. Consequently, it appeared that 84% of the 289 distinct tablets assessed in this study—those with size available—should be inappropriate for patients with swallowing disorders, representing 15.6% of the evaluations of tablets.

By reviewing studies on the effect of the SODF size on acceptability, the US Food and Drug Administration (FDA) observed that increasing the size of tablets and capsules is associated with an increase in the number of complaints related to swallowing difficulties [24]. According to their guidelines on “size, shape and other physical attributes of generic tablets and capsules”, medications greater than approximately 8 mm in diameter seem to be more likely to be related to swallowability issues [24]. Focusing on patients with swallowing disorders, it would seem logical that the threshold presented herein is smaller. However, another study which used a questionnaire with a printed diagram of tablets of varying sizes and shapes reported that tablets with sizes of 11 and 13 mm were perceived by the majority of older adults with dysphagia to cause difficulties in swallowing [16]. Such variations in the threshold of sizes may be explained by differences between people’s beliefs/perceptions and medicines use in real-life conditions. We assume that some patients could have overestimated their ability and willingness to swallow medium sized tablets, while healthcare professionals who mainly conducted the preparation and administration of medications in institutions could have underestimated their patients’ capacities, leading to modifications prior to administration, as well as other negatively connoted events and behaviors. In addition, although size appears to be a crucial parameter for the acceptability of SODFs, shape should also be investigated as it is likely to influence swallowability and consequently, acceptability. Variations in shape may be quantified comparing the largest cross sectional areas of the SODF [24]. Such physical measurements are rarely provided in the product’s common technical document (CTD), therefore samples will be needed for further research. Other attributes, such as smell, texture, and surface coating, could also influence acceptability [25,26]. According to Overgaard et al., the ideal tablet is small and white, with a strongly arched circular form, and is coated [27]. This collection of attributes highlights the relevance of a multivariate approach to investigate and objectively demonstrate the influence of a variety of such factors. Using a stated-preference method, a recent study revealed variations in adult outpatients’ preferences and willingness to pay regarding some physical attributes of SODFs [28].

In hospitals or care homes, healthcare professionals commonly select a dosage form among those available at their institution without a relevant decision support tool. Moreover, variations in regulatory constraints or prescribing systems and different local practices may limit choices, and at times exclude the possibility of prescribing the best dosage form regarding a patient’s dysphagia. In such cases, prescribers may be forced to resort to an inappropriate dosage form. Indeed, some patients still able to swallow SODFs, even two capsules in a single sitting, could receive a less accepted powder for oral solution [20]. The recourse of oral liquid preparations is a widely used alternative to SODFs for inpatients with dysphagia, even though their acceptability appeared to be suboptimal [21]. Orodispersible tablets (ODTs) seem to be a suitable option for those patients [20]. However, further investigations are needed to better understand the ODT acceptability drivers, as palatability may be an issue and some dysphagic patients could experience difficulty coordinating swallowing during the tablet’s disintegration. Results from the current study show that small tablets should also be considered as an appropriate formulation to treat these patients.

It should be noted that according to Fields et al., among patients that blame the size of SODFs, three-quarters experienced difficulties in taking tablets that were too large, while a quarter complained that tablets were too small [15]. In institutions, the preparation and administration of medications are primarily conducted by nurses and trained healthcare staff, which is not necessarily the case at home. Consequently, some handling issues may arise with smaller tablets, especially for the aging population living in their own home, resulting in small tablets being poorly accepted. Adequate devices and containers will be essential to alleviate these issues [24]. On another note, difficulty with administration in long term care might extend beyond the ability to swallow any given SODF. Indeed, both physical and psychological dimensions are of great importance, both of which may be equally affected by comorbidities. For instance, there are many patients, especially those with psychiatric disorders, for whom crushing tablets or opening capsules in order to mask the drug in food remain regrettably unavoidable to achieve administration if there is no viable alternative to the SODF. In such cases, there is a need for clear guidance in the product information to ensure treatment effectiveness and patient safety. Indeed, the SmPC made no mention of any such practices—i.e., tablet crushed—for 53 of the 62 distinct medicinal products that were modified prior to administration in this study. In only four cases did the SmPC explicitly permit crushing tablets for any patients unable to swallow the tablet(s) whole, while in a fifth case this modification was permitted for children of < 6 years of age. Finally, two SmPC stated that tablets should not be chewed, and in two further SmPC crushing tablets is only specifically contraindicated for pregnant women. These points underline the complexity of acceptability, which is driven by all the different features of the product, from size to the administration device, as well as the characteristics of different patients, with each administration occurring in a specific context. This may limit the generalizability of these findings. While this study has focused on how tablet size affects acceptability in older patients with a swallowing alteration in hospitals or care homes, more diversified sources of data (e.g., evaluations at home) and further data stratification by the type of patient or product will be needed to thoroughly encompass the multi-faceted concept of acceptability in future studies.

The many aspects of acceptability should be considered to select an appropriate dosage form. Such consideration is of the utmost importance to provide safe and successful care in the older population. Providing reliable evidence to healthcare professionals in order to assist them in choosing a suitable dosage form is thus essential.

## Figures and Tables

**Table 1:** Observer-reported outcomes for the tablet intake of patients with swallowing disorders according to the size of the tablets.

	Tablets ≥ 6.5 mm (*n* = 105)	Tablets < 6.5 mm (*n* = 36)	Statistical Test
**Result intake**			
fully taken	91 (89) ^a^	35 (97)	F ^b^: *p* = 0.47
partly taken	10 (10)	1 (3)	
not taken	1 (1)	0 (0)	
	*md ^c^*: *3*		
**Patient reaction**			
positive	5 (5)	0 (0)	F: *p* = 0.016
neutral	69 (67)	32 (91)	
negative	29 (28)	3 (9)	
	*md*: *2*	*md*: *1*	
**Preparation and administration time**			
short time	36 (35)	17 (47)	χ^2 d^: *p* = 0.09
medium time	38 (37)	15 (42)	
long time	30 (29)	4 (11)	
	*md*: *1*		
**Divided dose**			
use divided dose	31 (30)	2 (6)	χ^2^: *p* = 0.007
**Food/drink**			
use food/drink	62 (59)	20 (56)	χ^2^: *p* = 0.86
**Restraint**			
use restraint	7 (7)	0 (0)	χ^2^: *p* = 0.25
**Alteration**			
use alteration	54 (51)	8 (22)	χ^2^: *p* = 0.004
**Extra device**			
use device not provided	12 (11)	4 (11)	χ^2^: *p* = 1

^a^*n*(%): number and percentages; ^b^ F: Fisher’s exact test *p*-value; ^c^ md: missing data; ^d^ χ^2^: Pearson’s chi-squared test *p*-value.

## Supplementary Materials

The following are available online at https://www.mdpi.com/1999-4923/12/8/746/s1, Table S1: Characteristics of the patients and products for 936 evaluations of tablet intake, depending on swallowing alteration, Table S2: Size and shape of the 78 medicinal products formulated as a tablet that were assessed in patients with swallowing disorders, Table S3: Characteristics of the patients and products for 141 evaluations of tablet intake in patients with swallowing disorders, depending on the size of the tablets.
